# Communication with young people in paediatric and adult endocrine consultations: an intervention development and feasibility study

**DOI:** 10.1186/s12902-017-0182-6

**Published:** 2017-06-15

**Authors:** J. Downing, H. Gleeson, P.E. Clayton, J.R.E. Davis, P. Dimitri, J. Wales, B. Young, P. Callery

**Affiliations:** 1 Institute of Translational Medicine, University of Liverpool, Liverpool, UK; 2 Department of Endocrinology, Queen Elizabeth Hospital, Birmingham, UK; 3 School of Medical Sciences, University of Manchester, Royal Manchester Children’s Hospital, Central Manchester University Hospital’s Foundation Trust, Manchester Academic Health Science Centre, Manchester, UK; 4 Sheffield Children’s Hospital, Sheffield, UK; 5 University of Queensland and Department of Endocrinology & Diabetes, Lady Cilento Children’s Hospital, South Brisbane, Australia; 6 Institute of Psychology, Health and Society, University of Liverpool, Liverpool, UK; 7 School of Health Sciences, University of Manchester, Manchester Academic Health Science Centre, Manchester, UK; 8 Division of Nursing, Midwifery & Social Work, School of Health Sciences, University of Manchester, Jean McFarlane Building, Oxford Road, Manchester, M13 9PL UK

**Keywords:** Communication, Transition to adult care, Physician-patient relations, Endocrine system diseases, Feasibility studies

## Abstract

**Background:**

Communication is complex in endocrine care, particularly during transition from paediatric to adult services. The aims of this study were to examine the feasibility of interventions to support young people to interact with clinicians.

**Methods:**

Development and evaluation of a complex intervention in 2 phases: Pre-intervention observational study; Intervention feasibility study.

Purposive sample of recordings of 62 consultations with 58 young people aged 11–25 years with long-term endocrine conditions in two paediatric and two adult endocrine clinics.

Proportion of time talked during consultations, number and direction of questions asked; Paediatric Consultation Assessment Tool (PCAT); OPTION shared decision making tool; Medical Information Satisfaction Scale (MISS- 21).

Young people were invited to use one or more of: a prompt sheet to help them influence consultation agendas and raise questions; a summary sheet to record key information; and the www.explain.me.uk website.

**Results:**

Nearly two thirds of young people (63%) chose to use at least one communication intervention. Higher ratings for two PCAT items (95% CI 0.0 to 1.1 and 0.1 to 1.7) suggest interventions can support consultation skills. A higher proportion of accompanying persons (83%) than young people (64%) directed questions to clinicians. The proportion of young people asking questions was higher (84%) in the intervention phase than in the observation phase (71%).

**Conclusions:**

Interventions were acceptable and feasible. The Intervention phase was associated with YP asking more questions, which implies that the availability of interventions could promote interactivity.

## Background

Communicating with young people in endocrine care is complicated by the complexity of endocrine conditions and the need to address intimate matters that affect personal identity such as the medical management of growth, puberty and reduced fertility. As well as the demands associated with communication about sensitive issues, some conditions affect hearing [1] and non-verbal communication [2, 3].

The transition from child to adult changes the way that patients, parents, other carers and clinicians communicate with each other. Previous studies have focused on preparation of young people for increasing independence in self-management and the organisation of transfer between paediatric and adult services, including independent consulting [4, 5]. Young people must adapt to changes in the dynamics of communication as they develop increasing independence through adolescence [6–9]. Children can have little involvement in paediatric consultations [10–12], limiting their opportunities to learn how to talk with, question and make decisions with clinicians. Young people experience problems with communication in health care and can be reluctant to raise personal or sensitive issues or to ask questions that reveal poor adherence [13]. Disengagement from health care can result in young people with endocrine conditions not attending consultations following transfer to adult endocrine care [14, 15], with potential for increased morbidity and mortality [16, 17].

There is evidence that interventions can influence communication behaviours in health care consultations [18]. One potentially promising intervention involves various approaches to support question asking by patients, including question prompt sheets, with or without additional coaching, other written materials, audio and video recordings, and a computer program. Such support has shown some positive effects but overall the increase in questions asked was small [19]. Interventions to promote patient engagement can range from simple information provision, through patient activation, to patient–provider collaboration, each making different demands for staffing and adjustment of existing.practices [20]. However, there has been limited adaptation of interventions for young people. There is a lack of evidence to guide development of communication interventions that can be feasibly applied in endocrine consultations across adolescence and young adulthood.

## Methods

### Aims and objectives

The aims of this study were:

1. to examine communication behaviour between healthcare professionals and young people with endocrine conditions during adolescence and young adulthood, by assessing: young people’s active interactivity engagement in consultations; the quality of communication, young people’s involvement in decision making and satisfaction with consultations;
2. to examine the feasibility of interventions to promote communication including the take up and use of interventions offered, and to explore the use of observational outcome measures.

### Design

The design was a development and feasibility study [21]. In the development (pre-intervention) phase analysis of observed consultations provided the basis for development of interventions in consultation with young people and parents. In the feasibility (intervention) phase a further group of consultations was observed, including with participants who chose whether or not to use one or more of the interventions offered to them.

### Settings and participants

The setting was endocrine clinics at two paediatric and two adult regional centres between 2011 and 2014 serving a population with diversity of ethnicity, socio-economic status and urban/rural residence. Purposive samples were recruited in both phases to include patients of both genders across adolescence and young adulthood (defined as 11–25 years [22]) with Congenital adrenal hyperplasia (CAH), Hypopituitarism (HP), Turner syndrome (TS) or other long-term endocrine conditions including childhood cancer survivors.

Consultations were video recorded to include the young person, clinician and up to three companions. Other visitors or observers, and physical examination areas within the consultation room were audible but not visible on recordings. Participants who did not wish to be video recorded were offered the option of audio recording. The young person was instructed on how to stop or pause recording at any time if they wished. After recording they were given opportunities to identify any sections they wished to be removed.

In the pre-intervention phase consultations were analysed to provide the basis for development of interventions to improve communication. The method of development was consistent with principles of experience based design [23]. An advisory group consisting of young people, parents, clinicians and researchers met to review findings from consultation recordings and interviews described below and identified the three areas of intervention (pre-, during and post-consultation). The advisory group agreed that the intervention should support young people who wished to take a more active role in their consultations, for example by asking questions. It also identified the importance of flexibility to meet the individual needs of young people across the adolescent phase of development, and integrating the intervention within routine practice. A web based resource with downloadable tools for use in clinic and at home was identified as the most appropriate medium for these purposes. Draft interventions were developed and revised in two working groups with researchers, 7 young people and 7 parents.

The package of interventions could be used alone or in combination and comprised:

- Pre-consultation support to prepare questions: “Your Issues” - a prompt sheet for young people identify topics and questions to discuss in their consultation
- explain.me.uk a website with animations to support clinician explanations during consultations and/or for young people to view at home.
- Post –consultation: “Take Home Messages” – a summary sheet for young people and/or their parents and clinician to record answers and other key information; the explain.me.uk animations and supporting text for young people to review and to log information and generate an individual growth chart; a copy of the recording of the patient’s consultation.

Once the interventions were developed we observed their use during routine consultations. Young people were mailed an invitation to use the interventions, including a copy of the Your Issues/Take Home messages sheet and the explain.me.uk website address. On attendance consent and assent as appropriate were obtained and young people were again offered the sheet and an opportunity to review the website prior to their consultation. Clinicians were invited to have the website open on their clinic computer and young people were invited to use the prompt and summary sheetduring consultations. Use of interventions was identified during analysis of consultation recordings, and by self report during post-consultation follow up interviews.

Consultation recordings were transcribed and entered into NVivo 9 software [24]. The original recordings were synchronised with the transcripts to enable both to be reviewed during analysis.

### Outcomes

Aim 1 outcomes:

- Proportion of consultation time that young people, parents and clinicians talked. Transcripts were marked to identify the start and finish time of each utterance and attribution to young person, accompanying person/s, clinician/s and other (e.g. silence).
- Number of questions asked by young people, parents and companions identified from the consultation transcripts and coded by one coder and checked for agreement by a second coder. Questions were defined broadly by syntactical form rather than semantic content [25] and thus included all question forms (not restricted to requests for information) about any topic. This meant that any utterance that could be transcribed with a question mark was included, making the approach inclusive and maximising reliability by avoiding the need for interpretation. The direction of questions to a clinician, parent or other companion was identified from non-verbal as well as verbal cues in video recordings by two independent coders.
- Two raters independently viewed and scored each recording using: the Paediatric Consultation Assessment Tool (PCAT) a reliable and valid instrument developed to assess clinicians in triadic consultations with patients and parents [26] and OPTION, a reliable instrument that assesses to what degree patients are observed to be involved in shared decision making [27, 28]

Aim 2 outcomes:

Four key items of the PCAT instrument were identified as relating specifically to the objectives of the communication interventions developed from the findings of the pre-intervention phase: identifying reasons for the consultation; using skills which aided recall and understanding; screening and negotiating the agenda; tailoring the information. These were selected for analysis to examine whether there were differences in these items in addition to analysis of overall average scores in consultations by young people who used or did not use any of the interventions offered.

Patient satisfaction was self-reported after the consultation on the Medical Information Satisfaction Scale (MISS), a 21 item, seven-point Likert scale to assess patient satisfaction with consultations validated in UK patients in primary care [29]. The question “After talking with the doctor, I have a good idea of how long it will be before I am well again” was removed because early in data collection young people identified that it was inappropriate for their lifelong endocrine conditions. Negative responses were reverse coded and missing values were imputed using the mean of the relevant subscale [30]. The “Distress relief” scale to which this item contributed remained reliable (Cronbach’s alpha .77).

### Analysis

In keeping with the feasibility study design the analysis explored if differences between groups were consistent with the aim of promoting YPs’ interactivity in consultations rather than to detect significant effects. Allocation to intervention was by self-selection. The principle of “intention to treat” informed our approach of comparing the groups of young people observed in the pre-intervention phase, with those in the intervention phase, whether or not they chose to use interventions.

Scores on MISS-21, PCAT and OPTION were compared between consultations with and without intervention use via 95% confidence intervals for differences in means. Means for MISS-21 were compared between pre-intervention consultations, consultations with intervention use and consultations without intervention use using analysis of variance. As the study was not powered to detect significance, the test results were interpreted with caution. Data were analysed in IBM SPSS Statistics v20 [31].

#### Ethical considerations

Patients or their parents were initially approached by a consultant either face to face, via telephone or letter. Following a positive response patients and/or parents had the opportunity to discuss participation further with the study researcher. Verbal consent was then obtained on attendance for a consultation. Informed voluntary consent was obtained from participants or parents with informed assent from participants under 16 years. Participants could withdraw at any time. The study was approved by a Local Research Ethics Committee (reference number:10/H1017/18).

## Results

**Fig. 1:**
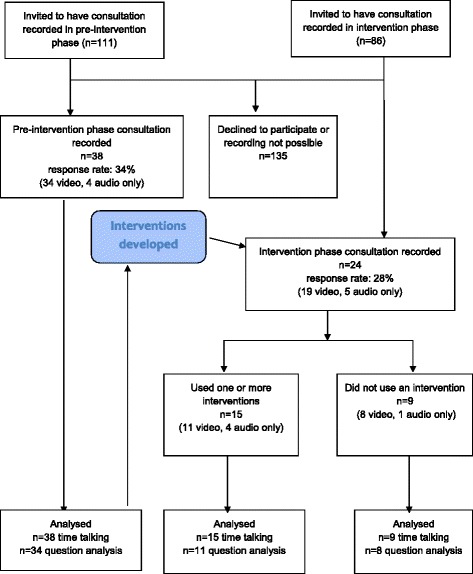
Flow Chart

A total of 38 consultations were recorded in the pre-intervention phase (4 by audio only) and 24 in the intervention phase (5 by audio only) (Fig. 1 Flow Diagram). Four young people participated in both the pre-intervention and intervention phases. There were more females, more participants under 16 and fewer with diagnoses other than CAH, HP or TS in the intervention phase compared to the pre-intervention phase (Table 1). Most (90%) were accompanied, usually by parents, in a few cases by another relatives or a friend. Twelve clinicians from paediatric and adult endocrine services were involved in consultations. Most consultations were routine reviews with a single clinician already known to the young person but 4 were first visits to a joint transition clinic (2 in each stage). The median consultation duration was 18.4 min (range 6.2–43.4, SD 7.9).

**Table 1:** Characteristics of participants by study group

Characteristic	Pre-intervention phase	Intervention phase		
		No interventions used	One or more interventions used	Total
Gender				
Male	18 (47%)	4 (80%)	1 (20%)	5 (21%)
Female	20 (53%)	5 (26%)	14 (74%)	19 (79%)
Age group				
11–13	8 (21%)	1 (11%)	3 (20%)	4 (17%)
14–16	12 (32%)	6 (67%)	6 (40%)	12 (50%)
17–19	12 (32%)	2 (22%)	4 (27%)	6 (25%)
20–25	6 (16%)	0	2 (13%)	2 (8%)
Diagnosis				
Congenital adrenal hyperplasia	6 (16%)	3	3	6 (25%)
Hypopituitarism	15 (39%)	5	3	8 (33%)
Turner syndrome	8 (21%)	1	8	9 (38%)
Other^a^	9 (24%)		1	1 (4%)
Total	38 (100%)	9 (100%)	15 (100%)	24 (100%)

^a^Other includes: 6 late effects of cancer; 2 rare endocrine conditions; and 2 isolated growth hormone deficiency

The young people who participated in both pre-intervention and intervention phases were: 2 females aged 11–13 with Hypopituitarism, 1 of whom used an intervention; and 2 males aged 14–16 years, 1 with HP who used an intervention and 1 with Congenital adrenal hyperplasia who did not use an intervention

In 7 pre-intervention and 3 intervention consultations the young person was meeting the clinician for the first time

### Take up of interventions

In the intervention phase the “Your Issues/Take Home Messages” sheet and explain.me.uk website were offered to 24 young people and 15 used at least one of these interventions. Clinicians were observed to refer to the explain.me.uk website during 6 consultations. The mean duration of all consultations was 20 min (range 10–44) and was similar in consultations with intervention use (21 min).

### Time talking

Young people’s talk accounted for a mean of 17% (median 19%) of the pre-intervention consultations. Their proportion of talk increased from 8% of consultation time in the 11–13 age group to 28% in the 20–25 age group. In the younger age groups (11–13 and 14–16) most talked for a lower proportion of time than parents (or others accompanying) but the proportion of young people talking more during consultations than parents increased with each age group and all the 20–25 year olds talked as much or more than anyone accompanying them. However, there were variations between the proportion of time individual young people talked in each age group, and the difference between least and most talking by young people increased in each successively older age group. These patterns were broadly similar in the intervention phase, with no evidence of an increase in the proportion of time talked associated with intervention use (Table 2).

**Table 2:** Young people’s talk as a percentage of consultation time by age group

		Pre-intervention phase		Intervention phase					
		Young person talk		Young person talk using any communication intervention			Young person talk using no communication intervention		
Age Group	*N*	Mean (Range)	Equal or more than a parent	*N*	Mean (Range)	Equal or more than a parent	*N*	Mean (Range)	Equal or more than a parent
11–13	8	8% (2–16%)	1 (13%)	3	6% (4–8%)	0	1	6%	0
14–16	12	13% (5–23%)	4 (33%)	6	14% (4–33%)	2 (33%)	6	19% (7–32%)	3 (50%)
17–19	12	21% (13–41%)	9 (75%)	4	22% (13–43%)	1 (25%)	2	24% (11–38%)	2 (100%)
20–25	6	28% (11–43%)	6 (100%)	2	31% (19–43%)	2 (100%)	0	n/a	n/a
All	38	17% (2–43%)	20 (53%)	15	17% (4–43%)	5 (33%)	9	19% (6–38%)	5 (56%)

### Question asking

Engagement as indicated by question asking also varied widely. Young people asked no questions at all during 13 (29%) consultations. In the remaining 40 consultations the number of questions asked by young people ranged from 1 to 5 at 11–13 years to 2–20 at 20–25 years (Table 3). There were wide variations in the number of questions directed to clinicians by young people (range 1–20 in the pre-intervention phase and 1–18 in the intervention phase). In the pre-intervention phase young people directed questions to clinicians in 62% (*n* = 21) of consultations. A higher proportion of young people who used at least one intervention 73% (*n* = 8) directed questions to a clinician than young people who did not use an intervention (63%, *n* = 5). More accompanying relatives also directed questions to clinicians if an intervention was used (Table 4).

**Table 3:** Young people asking questions by age group

Age group	Asked no question			Asked a question			Range of number of questions asked		
	Pre-intervention phase	Intervention phase		Pre-intervention phase	Intervention phase		Pre-intervention phase	Intervention phase	
		Any intervention used	No intervention used		Any intervention used	No intervention used		Any intervention used	No intervention used
11 to 13	3	0	0	4	1	1	1–5	1	1
14 to 16	4	2	0	7	4	5	1–5	3–6	2–17
17 to 19	3	0	0	8	2	2	1–16	4–10	2–4
20 to 25	0	1	n/a	5	1	n/a	2–20	0–6	n/a
All	10	3	0	24	8	8	1–20	0–10	1–17
	29%	16%		71%	84%

**Table 4:** Questions directed to clinicians

Questioner	Pre-intervention phase	Intervention phase		Total
	(*n* = 34, 4 unaccompanied)	Used an intervention (*n* = 11, 2 unaccompanied)	Did not use an intervention (*n* = 8, none unaccompanied)	(*n* = 53, 6 unaccompanied)
Young person	21 (62%) asked 77 questions (median = 2, range 1–20)	8 (73%) asked 22 questions (median = 2.5 range 1–6)	5 (63%) asked 33 questions (median = 4 range 2–18)	34 (64%) asked 132 questions (median = 2.5 range 1–20)
Accompanying relative (usually parent)	25 (83%) asked 112 questions (median = 4, range 1–11)	8 (89%) asked 59 questions (median = 6, range 1–22)	6 (75%) asked 39 questions (median = 4.5, range 1–18)	39 (83%) asked 210 questions (median = 4, range 1–22)

### Communication quality

The inter-rater reliability of the PCAT instrument was assessed by the Intra Class Correlation (ICC) where parents were present. For the whole sample the ICC was 0.46 (95% CI .19 to .66). The PCAT was designed for paediatric consultations but remained reliable for consultations with young people aged over 16 years where parents were present (ICC .67, 95% CI .26 to .87).As the PCAT was developed to score triadic consultations we confine our inter-rater reliability reports to the overall PCAT child and parent score.

The communication behaviours of clinicians assessed by the PCAT instrument were rated highly during consultations (overall mean 5.3 for young people and 5 for parents, scale 1–7, and consistent across sub-scales). The scores were also similar between consultations with communication intervention users for overall average PCAT scores or for two of the 4 PCAT items hypothesised to be most likely to be influenced by the interventions (‘screening and negotiating the agenda’, ‘tailoring the information’). However consultations in which interventions were used had higher scores for “identifying reasons for the consultation” (5.8 vs 5.2, 95% CI for difference in means 0.0 to 1.1) and “using skills which aided recall and understanding” (5.9 vs 5.0, 95% CI 0.1 to 1.7).

### Decision making

Although the inter-rater reliability of overall OPTION rating of consultations was adequate for parents (Cronbach’s alpha .73, ICC .55, 95% CI .25 to .76) and patients (Cronbach’s alpha .64, ICC .41, 95% CI .11 to .64)) there was poor agreement for specific items. In view of this lack of reliability we do not report OPTION scores.

### Satisfaction

Satisfaction with consultations as reported by the MISS responses was consistently high with a mean score of 5.9 (scale 1–7). However, young people using the intervention tools scored their comfort in communicating (mean scores 5.5 v 6.5, 95% CI for difference −1.9 to 0.0) and their rapport (5.6 v 6.5, 95% CI −1.6 to −0.2) with their doctor lower than those young people not using the intervention tools (Table 5). MISS responses indicate that those with pre-existing lower comfort and rapport chose to use an intervention, although it is also possible that these findings are due to a negative effect of the interventions on satisfaction.

**Table 5:** Satisfaction with consultation (MISS-21) by study group

MISS-21 scale	Pre-intervention phase	Intervention phase		
	(*n* = 34)	No interventions used (*n* = 9)	One or more interventions used (*n* = 13)	
	Mean (SD)	Mean (SD)	Mean (SD)	95% CI for difference in means
Distress relief	5.8 (0.9)	6.3 (0.8)	5.9 (0.7)	−.2 to 1.1
Rapport	6.1 (0.7)	6.5 (0.6)	5.6 (0.9)	.2 to 1.6
Comfort communicating	6.0 (0.9)	6.5 (0.6)	5.5 (1.2)	.0 to 1.9
Compliance intent	5.6 (1.2)	6.2 (0.9)	5.8 (1.0)	−.5 to 1.2

Comparison of all three groups: Distress relief ANOVA F = 1.27, df = 2 and 49, p = .291; Rapport ANOVA F = 2.08, df = 2 and 53, p = .028; Comfort communicating ANOVA F = 2.77, df = 2 and 52, p = .072; Compliance intent: ANOVA F = 1.25, df = 2 and 53, p = .294

## Discussion

### Young people’s engagement in consultations

The time young people talked in consultations and the number of questions they asked varied widely. The proportion of time that young people talked according to age groups 11–13 (8%) and 14–16 (13%) was comparable with studies of the time talked by children and young people aged up to 15 years of age in a range of paediatric outpatient and general practice settings (2–14%) [10] and the proportion of utterances made by children aged 5–17 in various paediatric subspecialty visits (15%) [32]. Parents talked more than young people in two thirds of consultations of 14–16 year olds. Thus young people have limited space in consultations in which to hone the skills of talking with clinicians, either because they choose to talk less than parents, or they are given less opportunity. Although there was an average increase across the age ranges, the range between the lowest and highest proportion of talking by young people widened in the older age groups. Our definition of questions was by syntax and therefore broad: it included questions that were not necessarily requests for information [25]. Even with this inclusive definition 13 (Table 3) young people asked no questions at all. There was a wide variation in questions asked as well as time talked. The relationship between consultation duration and question asking is uncertain – more questions might be expected to extend consultations, and longer consultations to give more opportunities for questions to be asked. The variations in time talked and question asking indicate that differences in the degree of active participation in consultations emerged in middle adolescence and persisted in young adults. Questioning has been conceptualised by researchers as a more ‘participative’ style of health consultation behaviours and asking fewer questions as a more ‘passive’ style [33]. Individuals may prefer to adopt different roles and styles of communication in consultations. However, some young people may need additional support to ask questions and talk in consultations.

### Potential for intervention

Communication with health professionals can be improved by interventions [18] including prompt sheets to support asking questions in consultations [19]. However these interventions have only been designed for adults. The study examined the use of interventions designed to support young people during their transition from paediatric to adult care and to help them to develop as effective participants in consultations in adult services. The ‘Your Issues’ sheet was developed to help young people to identify agenda items and to formulate questions to ask in consultations. On its reverse was the ‘Take Home Messages’ sheet designed to support summing up and to provide young people with a record of key information to take from consultations. The ‘Your Issues/Take Home Messages’ sheets were supported by the www.explain.me.uk web site, to help clinicians answer questions and provide information in clinics, and for young people to review and keep their own personalised record. Nearly two thirds of the young people (63%) took up the offer to use at least one of these communication interventions, and in the intervention phase a higher proportion of young people who used at least one intervention directed questions to a clinician than young people who did not use an intervention. The results should be interpreted with caution because the sample sizes in the intervention phase were small and the study was not designed to test hypotheses. However, these findings are consistent with interventions supporting young people to direct questions to clinicians.

### Outcome measures

It was not possible to assess decision making reliably using the OPTION tool, which is likely to be due to the difficulty of identifying discrete decisions in these routine follow up consultations. The PCAT scoring was more reliable. As the PCAT measures clinician behaviour, the higher scores for sub-scale PCAT items (“identifying reasons for the consultation” and “using skills which aided recall and understanding”) in consultations with intervention users, suggest that interventions could contribute to changes in consultation behaviour which support these activities. This highlights the potential need for interventions to support clinicians to promote changes in communication behaviour.

Although satisfaction as measured by the MISS was generally high, communication intervention users reported lower ratings for comfort in communicating and rapport. This could suggest that young people chose to use the interventions because they had less rapport and were less comfortable communicating with clinicians. However, it is also possible that interventions could affect satisfaction negatively, for example if clinicians spent increased time looking at computers in consultations [34], or because writing on prompt sheets such as the Your Issues/Take Home Messages could inhibit the informality and flexibility of interactions.

#### Strengths and limitations

This is the first study of which we are aware to analyse young people’s engagement in consultations using video recordings with the exception of a study of emotional cues of adolescent cancer survivors [35]. The inclusion of young people across the age range 11–25 enabled us to observe changes in communication behaviours between young people who were at different stages of adolescence and young adulthood and the transfer from paediatric to adult care. Young people in our sample talked for an increasing proportion of consultations in each successive age group.. This did not appear to be altered by interventions and as the process of measurement was laborious it may have limited use as an outcome measure. The analysis of the number and direction of questions asked provided insight into the potential for interventions to support young people’s interactivity in consultations. Future studies would benefit from examining question asking and direction as an outcome. Our study has limitations. The observational design could not test the effectiveness of the interventions definitively and our data do not compare individuals pre and post intervention. We were not able to assess the distribution of learning difficulties in the sample, although participants were only recruited if they could give informed consent or assent. We observed users during their first exposure to the interventions and sustained use and practice may be required to change young people’s behaviour and interactions with clnicians in consultations. It was apparent that most of the young people were attending follow up consultations for conditions which had been managed for some time. However, it was also clear that the nature of consultations varied, for various reasons, including: the current status of the young person’s health; management priorities and concerns; the young person’s own development and role in managing their condition and in consultations vis a vis their parents. This highlights the need to consider the context of consultations when developing and evaluating interventions. Young people may find different components of an intervention package more relevant to their needs at different times in their development.

The PCAT instrument could not be used to assess dyadic consultations with unaccompanied young people. One question of the MISS-21 was inappropriate for an endocrine population. This highlights the lack of instruments designed specifically for study of adolescent consultations, and endocrine conditions, and more generally the problem of assessing communication from the patient perspective.

## Conclusions

Increasing interactivity in consultations has potential long term benefits as young people go through the transition to adult self care of their endocrine conditions. Our study has identified how some young people can have limited involvement in consultations and this can persist into young adulthood. This indicates a need to offer young people support in communicating with clinicians. As part of this study we developed a package of interventions that aimed to promote young people’s interactivity in consultations with health professionals in endocrine clinics. The majority of young people to whom we offered the interventions, were willing to try at least one. This study demonstrated the feasibility of using the intervention package. However, further research is required both to test the effectiveness of interventions definitively and to establish for which young people the interventions have most value.

## Abbreviations

CAH: Congenital adrenal hyperplasia
HP: Hypopitutarism
MISS- 21: Medical information satisfaction scale
PCAT: Paediatric consultation assessment tool
TS: Turner syndrome
YP: Young people
